# Craving for Ketamine and Its Relationship With Clinical Outcome Indicators in Males With Ketamine Use Disorder

**DOI:** 10.3389/fpsyt.2021.476205

**Published:** 2021-06-08

**Authors:** Peng-Wei Wang, Huang-Chi Lin, Tai-Ling Liu, Chih-Yao Hsu, Yu-Yi Yang, Hung-Chi Wu, Cheng-Fang Yen

**Affiliations:** ^1^Department of Psychiatry, Kaohsiung Medical University Hospital, Kaohsiung, Taiwan; ^2^Department of Psychiatry, Faculty of Medicine and Graduate Institute of Medicine, College of Medicine, Kaohsiung Medical University, Kaohsiung, Taiwan; ^3^Departments of Addiction Science, Kai-Suan Psychiatric Hospital, Kaohsiung, Taiwan

**Keywords:** ketamine, craving, DDQ, addiction, readiness to change, dependence level

## Abstract

**Background:** Craving is considered a hallmark of substance use disorder and is one of the criteria of substance use disorder. The Desires for Drug Questionnaire (DDQ) is a widely used questionnaire to assess craving for heroin. This study aimed to examine the psychometric properties of the ketamine version of the DDQ (DDQ-K) and the associations between craving for ketamine as measured using the DDQ-K and clinical outcome indicators in individuals with ketamine use disorder.

**Methods:** In total, 651 individuals with ketamine use disorder completed the DDQ-K and the Visual Analog Craving Scale (VACS). Demographic data, severity of ketamine use, money spent on ketamine, positive/negative aspects of ketamine use, and readiness to change ketamine use were also recorded. We examined the reliability (internal consistency), construct validity (factor structure), and concurrent validity of the DDQ-K. Multiple regression analysis was used to examine the relationships of craving measured using the DDQ-K with clinical outcome indicators, including money spent on ketamine, severity of ketamine use, positive/negative aspects of ketamine use, and readiness to change ketamine use.

**Results:** The original three-factor model of the DDQ-K was acceptable for use in individuals with ketamine use disorder according to confirmatory factor analysis. The subscales of Desire and Intention and Negative Reinforcement of the DDQ-K, but not the subscale of Control, were of acceptable concurrent validity. The score on the Desire and Intention subscale was positively associated with the level of ketamine dependence, money spent on ketamine use, and positive/negative aspects of ketamine use and negatively associated with readiness to change ketamine use.

**Conclusion:** This study supported the use of the Desire and Intention and Negative Reinforcement subscales of the DDQ-K to assess craving in patients with ketamine use.

## Introduction

Craving is a subjective experience of people who are dependent on addictive substances, in the sense of their desire to use a substance (1). Craving often intrudes into the daily life of people with substance dependence, dominating their thoughts and provoking considerable distress. Previous studies showed that craving is closely related to substance use in people with substance dependence (2, 3). Beyond this important clinical concern, there are several potential clinical uses of craving assessment. First, craving is listed as a criteria of substance use disorder in the Diagnostic and Statistical Manual of Mental Disorders (DSM-5) and as a feature of psychoactive substance dependence in the 10th version of the International Classification of Diseases (ICD-10) (4, 5). Second, several studies have indicated that craving is significantly predictive of substance use relapse (6–8). Therefore, craving has the potential to predict prognosis. Third, reducing the level of craving has been reported as resulting in better treatment outcomes, with the exception of reduction in or elimination of addictive substance use (9).

The most common methods used to assess craving are a single-item Likert-type rating scale or the visual analog scale (VAS) in both clinical and research settings (10, 11). Despite advantages such as ease of use and good sensitivity to detect rapid change (12), single-item scales have been criticized in terms of their failure to reflect the presumably multidimensional nature of craving (13). In addictive substance users, craving may consist of components across multiple domains, such as emotional experiences, cognitive experiences, and psychophysiological experiences (1). Rosenberg (14) reported on an increasing number of studies aiming to develop multi-item self-reported questionnaires to assess craving for both legal and illicit substances.

The Desires for Drug Questionnaire (DDQ) is a widely used questionnaire for measuring instant and periodic craving (14). The DDQ was originally adapted from the Desire for Alcohol Questionnaire (DAQ) to assess heroin craving at the present time as a multidimensional motivational state by Franken et al. (15). The DDQ consists of three subscales: Desire and Intention, Negative Reinforcement, and Control. It has been shown to be of good reliability and validity for the assessment of craving in individuals with heroin use (15). Furthermore, the DDQ has been modified for use to assess craving for addictive substances other than heroin (16–18). However, the use of the DDQ to assess craving for ketamine has not been examined.

Ketamine, in which the main action of the substance is that of a non-competitive N-methyl-D-aspartate (NMDA) receptor antagonist, is a widely used anesthetic. Some of the most appealing effects reported by non-medical ketamine users include “melting into surroundings,” “out of body experience,” and “giggliness” (19). Furthermore, at large dosages, ketamine may result in a prominent dissociated experience, referred to as a “K-hole” (20). However, ketamine use may result in mental and physical illnesses (21, 22). Ketamine abuse can be traced back to the 1970s (23) and has been a commonly abused drug worldwide since the 1990s (24, 25), particularly in East Asia (26). Ketamine use increased rapidly in the 2000s (27), and it is now one of the most common addictive drugs used in Taiwan (26).

There has been no study on craving in ketamine users. Further study to examine the suitability of the DDQ for the assessment of craving in ketamine users is essential. The first aim of the present study was to examine the psychometric properties of the Chinese-Mandarin ketamine version of the DDQ (DDQ-K) in males with ketamine use disorder. The second aim was to explore the associations between the craving scores on the three subscales of the DDQ-K and clinical outcome indicators.

## Methods

### Participants

A total of 651 male ketamine users were enrolled into this study from a drug education center in South Taiwan. The inclusion criteria for these participants were (1) having ketamine use disorder and a positive urine ketamine test, (2) absence of other substance use disorders except tobacco use, and (3) absence of psychiatric diagnoses of schizophrenia, major depressive disorder, and bipolar disorder. The participants underwent interviews to collect baseline data, including age, sex, level of education, and money spent on ketamine use. The mean age of the participants was 25.5 years [standard deviation (SD): 6.43 years]. The institutional review board of Kaohsiung Medical University approved the study protocol.

### Assessments

#### The Chinese-Mandarin Ketamine Version of the Desires for Drug Questionnaire

The original DDQ, which consists of 13 items measured on a 7-point Likert scale ranging from 0 (totally disagree) to 6 (totally agree), is used to assess instant craving in heroin-dependent individuals (15). The DDQ is composed of three subscales: Desire and Intention (e.g., “My desire to use heroin now is overwhelming”), Negative Reinforcement (e.g., “Even major problems in my life would not bother me if I used heroin now”), and Control (e.g., “If I started using heroin now, I would be able to stop”). The Chinese-Mandarin version of the DDQ has been shown to be a reliable and valid tool for the measurement of craving in heroin-dependent individuals (28). The DDQ-K is a variant of the Chinese-Mandarin version of the DDQ; “heroin” is replaced with “ketamine,” and the scale measures craving for ketamine at the present moment. A higher total score indicates a higher level of craving for ketamine.

#### Visual Analog Craving Scale

The Visual Analog Craving Scale (VACS), modified from previous studies (29, 30), was used to measure the level of craving in ketamine-dependent individuals. The VACS consisted of the following single question: How much did you crave/desire/want to use ketamine in the preceding week? The level of craving was rated from 0 (not at all) to 100 (very much).

#### Chinese-Mandarin Version of the Severity of Dependence Scale

The Chinese-Mandarin Version of the Severity of Dependence Scale (SDS^ch^), which consists of five items, was used to assess the level of dependence of ketamine-dependent individuals in the preceding week (31). The score of the SDS^ch^ ranges from 0 to 15, with a higher score indicating more severe dependence.

#### Drug Use Disorders Identification Test-Extended

The Drug Use Disorders Identification Test-Extended (DUDIT-E) is a screening tool used to identify the history of problematic drug use (32). The DUDIT-E consists of three subscales: positive and negative aspects of ketamine use and readiness to change ketamine use. A high total score on each subscale indicates increased awareness of positive outcome expectancy and negative consequences of ketamine use and a high level of readiness to change ketamine use, respectively.

### Data Analysis

Confirmatory factor analysis was used to examine the three-factor model of the DDQ-K. Four indices, namely, the root mean square error of approximation (RMSEA), non-normed fit index (NNFI), standardized root mean square residual (SRMR), and comparative fit index (CFI), were used to assess the model fit (33). An RMSEA <0.10 was acceptable (34), while values lower than 0.08 for the SRMR and larger than 0.90 for the NNFI and CFI indicated a close fit.

The internal consistency of the three subscales of the DDQ-K was examined by Cronbach's alpha. Regarding concurrent validity, we used Pearson's correlation to examine the associations between levels of craving on the three subscales of the DDQ-K with craving on the VACS.

In order to examine the relationships of craving with clinical outcome indicators, we used a regression model to explore the associations of craving measured using the three subscales of the DDQ-K with the level of ketamine dependence, money spent on ketamine use, positive/negative aspects of ketamine use, and readiness to change ketamine use, controlling for the effects of age, education, and length of ketamine use. A two-tailed *p* < 0.05 was considered statically significant.

## Results

The demographic and ketamine-use characteristics are presented in Table 1. The fit indexes for the original three-factor model DDQ-K were acceptable (RMSEA = 0.066, SRMR = 0.037, NNFI = 0.936, CFI = 0.9577). The internal consistency (Cronbach's alpha) of the three subscales of Desire and Intention, Negative Reinforcement, and Control on the DDQ-K was 0.91, 0.85, and 0.82, respectively, indicating that the internal consistency of the three subscales was acceptable.

**Table 1:** Demographic and ketamine-related characteristics of male ketamine users.

	**Mean (SD)**
Age (years)	25.50 (6.43)
Education (years)	11.32 (2.36)
Length of ketamine use (years)	4.20 (10.53)
Money spent on ketamine use (NT/day)	517.24 (1175.35)
Craving for ketamine on the VACS	12.24 (21.14)
Do you think use of ketamine was out of control (Q1 on the SDS^ch^)	0.50 (0.84)
Did the prospect of missing a dose make you anxious or worried (Q2 on the SDS^ch^)	0.40 (0.71)
Did you worry about your use of ketamine (Q3 on the SDS^ch^)	0.63 (0.87)
Did you wish you could stop (Q4 on the SDS^ch^)	1.63 (1.21)
How difficult did you find it to stop or go without ketamine (Q5 on the SDS^ch^)	0.39 (0.72)
Level of ketamine dependence on the SDS^ch^	3.56 (3.11)
Craving for ketamine on the DDQ-K	
Desire and intention	1.04 (1.84)
Negative reinforcement	1.16 (1.95)
Control	5.56 (3.70)
Positive aspects of ketamine use on the DUDIT-E	10.08 (11.57)
Negative aspects of ketamine use on the DUDIT-E	14.03 (13.81)
Readiness to change ketamine use on the DUDIT-E	3.90 (2.38)

*DDQ-K, The Chinese-Mandarin Ketamine version of the Desires for Drug Questionnaire; DUDIT-E, Drug Use Disorders Identification Test-Extended; SDS^ch^, Chinese-Mandarin version of the Severity of Dependence Scale; VACS, Visual Analog Craving Scale*.

The results of examining the associations of craving levels on the three subscales of the DDQ-K with craving for ketamine use as measured by the VACS, money spent on ketamine use, level of ketamine dependence, positive and negative aspects of ketamine use, and readiness to change ketamine use are shown in Table 2. The scores on both subscales of Desire and Intention and Negative Reinforcement on the DDQ-K were positively associated with money spent on ketamine use, craving for ketamine use as measured by the VACS, level of ketamine dependence, and positive and negative aspects of ketamine use (Table 3). The scores on the subscales of Desire and Intention and Negative Reinforcement on the DDQ-K were also negatively associated with readiness to change ketamine use.

**Table 2:** Correlations between the three subscales of the DDQ-K and ketamine-related outcomes: Pearson's *r*.

	**Desire and** **intention**	**Negative** **reinforcement**	**Control**
Length of ketamine use	0.05	0.03	0.01
Money spent on ketamine use	0.63^***^	0.55^***^	−0.09^*^
Craving for ketamine on the VACS	0.57^***^	0.46^***^	−0.004
Q1 on the SDS^ch^	0.35^***^	0.29^***^	−0.11^**^
Q2 on the SDS^ch^	0.41^***^	0.36^***^	−0.07
Q3 on the SDS^ch^	0.30^***^	0.25^***^	0.06
Q4 on the SDS^ch^	−0.02	0.01	0.13^**^
Q5 on the SDS^ch^	0.37^***^	0.32^***^	−0.08
Level of ketamine dependence on the SDS^ch^	0.35^***^	0.31^***^	0.006
Positive aspects of ketamine use	0.43^***^	0.44^***^	−0.03
Negative aspects of ketamine use	0.39^***^	0.40^***^	−0.06
Readiness to change ketamine use	−0.14^***^	−0.09^*^	0.11^**^

* *p* < 0.05;

** *p < 0.01;*

*** *p < 0.001.*

*VACS, Visual Analog Craving Scale; SDS^ch^, Chinese-Mandarin version of the Severity of Dependence Scale*.

**Table 3:** Associations of subscales of the DDQ-K with money spent on ketamine, severity of ketamine dependence, attitudes toward ketamine use, and readiness to accept treatment: multiple regression analysis^a^.

	**Money spent on ketamine use**		**Level of ketamine dependence**		**Positive aspects of ketamine use**		**Negative aspects of ketamine use**		**Readiness to change ketamine use**	
	**Coefficient**	***p***	**Coefficient**	***p***	**Coefficient**	***p***	**Coefficient**	***p***	**Coefficient**	***p***
Desire and intention	364.32	<0.001	0.53	<0.001	1.35	0.001	1.56	0.005	–0.25	0.006
Negative reinforcement	41.48	0.214	0.06	0.568	1.52	<0.001	1.49	0.004	0.08	0.362

a *Controlled for the effects of age, education level, and length of ketamine use*.

Contrarily, the score on the Control subscale was negatively associated with money spent on ketamine use and positively associated with readiness to change ketamine use; however, both associations were weak. The score on the Control subscale was not significantly related to craving as measured using the VACS, level of ketamine dependence, or negative aspects of ketamine use.

The scores on the Desire and Intention and Negative Reinforcement subscales were assessed in terms of their relationships with ketamine use-related outcome indicators. The results indicated that after controlling for the effects of age, education level, and length of ketamine use, the scores on both subscales were positively correlated with the positive and negative aspects of ketamine use. In addition, the Desire and Intention subscale score, but not the Negative Reinforcement subscale score, was positively related to money spent on ketamine use and level of ketamine dependence but negatively related to readiness to accept treatment.

## Discussion

The results of the present study showed that the original three-factor model of the DDQ-K was acceptable for use to assess individuals with ketamine use disorder. The concurrent validity was acceptable for the subscales of Desire and Intention and Negative Reinforcement, but not the subscale of Control, as the scores on the former two subscales of the DDQ-K were positively associated with craving as assessed using the VACS. The results showed that both subscales of Desire and Intention and Negative Reinforcement on the DDQ-K were suitable for assessing craving for ketamine.

In the original version of the DDQ, the level of craving on the Control subscale was weakly correlated with the level of craving as measured using the VACS (15). The original concept for this subscale of the DDQ was that substance users may overestimate their self-control over their substance use because they crave the substance. However, in our study, the score on the Control subscale was not associated with the level of craving as measured using the VACS. A possible explanation for the inconsistency between our results and those of the previous study is that the score on the Control subscale of the DDQ-K reflects participants' perceived abilities to control their ketamine use, which may not represent their status of craving. Research has shown that craving is associated with activation of the nucleus accumbens, ventral tegmental area, and other reward regions (35, 36). However, the ability to control drug use is associated with the function of the prefrontal cortex, which regulates the ability to inhibit impulsive or habitual behavior (37) and allows flexible pursuit of long-term goals (35, 37). From the biological viewpoint, craving and self-control involve different brain circuits. This may support our result in terms of the level of craving according to the VACS and the score on the Control subscale on the DDQ-K not being associated with each other.

Furthermore, the level of control was negatively associated with money spent on ketamine use and positively associated with the level of readiness to change ketamine use. These results further indicated that the score on the Control subscale of the DDQ-K reflects ketamine users' perceived control abilities but not the level of craving measured in the original version of the DDQ. However, the correlation between the score on the Control subscale and money spent on ketamine use was weak. Chinese culture emphasizes self-control to maintain interpersonal and group harmony; therefore, people in a Chinese group-oriented society are required to control their desires (38). People who have difficulties in controlling their desires are considered immature and unsuccessful. This cultural value may influence ketamine users to assume that they have a full ability to control their ketamine use. Therefore, dissociation between self-perceived ability and behavior, which has been considered a characteristic of drug users (39), may develop among ketamine users in Taiwan. Moreover, substance users may have an unconscious cognition to restrict substance use that is not regulated by conscious control (40), meaning that restricting ketamine use may not solely involve conscious control.

The manifestation of craving as described in the DSM-5 is a strong desire or urge to use a drug (5). The results of multiple regression analysis indicated that only the score on the Desire and Intention subscale was significantly related to all clinical outcome indicators, supporting the concept of the DSM-5 that a strong desire or urge to use a drug is one phenotype of craving in substance use disorder. In addition, our results highlighted that measuring the desire to use ketamine is essential in clinical practice for individuals with ketamine use disorder.

It was interesting to find that the scores on the Desire and Intention and Negative Reinforcement subscales were related to positive and negative aspects of ketamine use. According to the Transtheoretical Model of Behavior Change (TTM), ketamine users who have high levels of craving as measured on the Desire and Intention and Negative Reinforcement subscales may have an ambivalent attitude toward ketamine use (41), which in turn may lead to ketamine users delaying taking action to change their ketamine use behavior. Furthermore, our results indicated that the score on the Desire and Intention subscale was negatively associated with readiness to accept treatment. These results further implied that a higher score on the Desire and Intention subscale of the DDQ-K may indicate less motivation to change ketamine use due to a more ambivalent attitude to drug use and less readiness to accept treatment.

There were several limitations of this study. First, craving was measured using a self-reported assessment, which may be influenced by factors such as a defensive attitude or subjects not assessing their internal state accurately (42). Despite these restrictions, self-reported questionnaires are mainstream practice for the assessment of drug users (36). Second, we did not examine whether participants used ketamine or not just before assessing their craving. Using, or not using, ketamine prior to completion of the questionnaire may affect the level of craving in the participants. Third, the participants were male and enrolled from the community. In addition, we did not enroll health participants as a control group. More studies are necessary to show that the DDQ-K can be used in different situations, for example, in female ketamine users and controlled environments. Fourth, the different time frames of craving assessment examined using the VACS (1 week) and the DDQ-K (current) is also important because recall of craving may be subject to a variety of biases (42). Fifth, the ketamine users may use ketamine to relieve their depressive symptoms (43). It warrants further study to explore the effect of dose of ketamine on depression.

## Conclusion

The original three-factor model of the DDQ-K was acceptable for the assessment of individuals with ketamine use disorder. Tiffany and Wray (36) suggested that a small set of self-reported questionnaires is a reliable and useful tool for measuring craving in clinical practice. The subscales of Desire and Intention and Negative Reinforcement, but not the subscale of Control, were suitable subscales of the self-reported DDQ-K questionnaire for use to assess craving for ketamine.

## Data Availability Statement

The raw data supporting the conclusions of this article will be made available by the authors, without undue reservation.

## Ethics Statement

The studies involving human participants were reviewed and approved by The Institutional Review Board of Kaohsiung Medical University approved the study protocol. The patients/participants provided their written informed consent to participate in this study.

## Author Contributions

C-FY organized this study team. H-CW design this study. P-WW did the analysis and wrote the paper. T-LL and C-YH did the data collection. H-CL and Y-YY did the analysis. All authors contributed to the article and approved the submitted version.

## Conflict of Interest

The authors declare that the research was conducted in the absence of any commercial or financial relationships that could be construed as a potential conflict of interest.
